# P05.53. Variations in the implementation and characteristics of chiropractic services in VA

**DOI:** 10.1186/1472-6882-12-S1-P413

**Published:** 2012-06-12

**Authors:** A Lisi, R Khorsan, M Smith, B Mittman

**Affiliations:** 1 Veterans Health Administration, West Haven, USA; 2 Samueli Institute for Information Biology, Corona del Mar, USA; 3 Sepulveda Research Corporation, Sepulveda, USA; 4 VA Center for Implementation Practice and Research Support, Sepulveda, USA

## Purpose

To document and assess cross-facility differences in the planning, implementation and current characteristics of chiropractic services in VA.

## Methods

Observational comparative case study approach using data collected via semi-structured key stakeholder interviews and abstracted from policy documents and other material. Six VA facilities delivering chiropractic services on-station were studied. Transcripts and documents were coded and analyzed via directed content analysis.

## Results

One hundred and sixteen stakeholder interviews and 75 source documents were analyzed. The six chiropractic programs varied in terms of methods and patterns of patient access (direct referral vs. triaged referral; length of wait time), chiropractor appointment type (employee vs. contract; full-time vs. part-time), level and nature of the clinic’s integration with other clinical programs and services (e.g., collaborative integration vs. parallel practice), and other features. Non-DC clinicians expressed diverse views on the appropriateness of chiropractic services, yet most were favorable. These clinicians displayed limited knowledge of the current evidence on managing non-operative spinal conditions and the components of chiropractic case management, however. VA facility staff identified professional and interpersonal attributes of the DC clinicians as important facilitators of clinic status and integration. Chiropractors varied in their professional attributes and self-perceived degree of successful integration. Other variations in planning, implementation and clinic features were identified.

## Conclusion

VA facilities varied in the manner in which they planned and implemented new chiropractic care programs and in the features of the clinics as established. The findings from this study and may offer policy and practice leaders important insights into effective strategies for managing new clinical program implementation. This study provides a framework for studying the introduction of new clinical services to VA and other healthcare systems.

